# Erwinia asparaginase (crisantaspase) increases plasma levels of serine and glycine

**DOI:** 10.3389/fonc.2022.1035537

**Published:** 2022-12-12

**Authors:** Dominique Bollino, J. Preston Claiborne, Kanwal Hameed, Xinrong Ma, Kayla M. Tighe, Brandon Carter-Cooper, Rena G. Lapidus, Erin T. Strovel, Ashkan Emadi

**Affiliations:** ^1^ School of Medicine, University of Maryland Marlene and Stewart Greenebaum Comprehensive Cancer Center, Baltimore, MD, United States; ^2^ Department of Medicine, School of Medicine, University of Maryland Marlene and Stewart Greenebaum Comprehensive Cancer Center, Baltimore, MD, United States; ^3^ Department of Pediatrics, School of Medicine, University of Maryland Marlene and Stewart Greenebaum Comprehensive Cancer Center, Baltimore, MD, United States; ^4^ Department of Pharmacology, School of Medicine, University of Maryland Marlene and Stewart Greenebaum Comprehensive Cancer Center, Baltimore, MD, United States

**Keywords:** asparaginase, acute myeloid leukemia, erwinase, pegcrisantaspase, amino acids, serine

## Abstract

The impact of asparaginases on plasma asparagine and glutamine is well established. However, the effect of asparaginases, particularly those derived from *Erwinia chrysanthemi* (also called crisantaspase), on circulating levels of other amino acids is unknown. We examined comprehensive plasma amino acid panel measurements in healthy immunodeficient/immunocompetent mice as well as in preclinical mouse models of acute myeloid leukemia (AML) and pancreatic ductal adenocarcinoma (PDAC) using long-acting crisantaspase, and in an AML clinical study (NCT02283190) using short-acting crisantaspase. In addition to the expected decrease of plasma glutamine and asparagine, we observed a significant increase in plasma serine and glycine post-crisantaspase. In PDAC tumors, crisantaspase treatment significantly increased expression of serine biosynthesis enzymes. We then systematically reviewed clinical studies using asparaginase products to determine the extent of plasma amino acid reporting and found that only plasma levels of glutamine/glutamate and asparagine/aspartate were reported, without measuring other amino acid changes post-asparaginase. To the best of our knowledge, we are the first to report comprehensive plasma amino acid changes in mice and humans treated with asparaginase. As dysregulated serine metabolism has been implicated in tumor development, our findings offer insights into how leukemia/cancer cells may potentially overcome glutamine/asparagine restriction, which can be used to design future synergistic therapeutic approaches.

## Background

Asparaginases are enzymes that catalyze the hydrolysis of asparagine and glutamine to aspartate and glutamate, respectively. With the first approval by the United States (U.S.) Food and Drug Administration (FDA) in 1978, asparaginases have since become an essential component of multi-agent chemotherapy for pediatric and adult acute lymphoblastic leukemia (ALL). Clinically available asparaginase is derived from two bacterial sources*: Escherichia coli *(*E. coli*) and *Erwinia chrysanthemi*, with asparaginase from *Erwinia* (also called crisantaspase) demonstrating higher glutaminase activity (1, 2). In the U.S. today, commercially available preparations of *E. coli* asparaginase include Oncospar (pegaspargase) and Asparlas (calasparagase pegol-mknl), both pegylated forms which prolong plasma retention (3). *Erwinia* asparaginases include Erwinaze (asparaginase *Erwinia chrysanthemi*) and Rylaze (asparaginase *Erwinia chrysanthemi* [recombinant]-rywn), which are indicated for use in patients that have developed hypersensitivity to *E. coli*-derived asparaginase. Outside of their well-established role in ALL, asparaginases also have an emerging role in acute myeloid leukemia (AML). In a phase I clinical trial in patients with relapsed or refractory (R/R) AML, Erwinaze was shown to effectively deplete plasma asparagine and glutamine without a dose limiting toxicity, which correlated to its anti-AML activity (4). Asparaginases are also being explored in the treatment of solid tumors, including pancreatic ductal adenocarcinoma (PDAC), that are sensitive to asparagine and glutamine depletion (5, 6).

Cancer cells undergo metabolic reprogramming that can make them dependent on exogenous asparagine and glutamine to sustain rapid cellular proliferation. Glutamine is critical for maintaining redox homeostasis through glutathione synthesis, for supplying nitrogen for pyrimidine synthesis, and for generation of nonessential amino acids for protein synthesis (7, 8). Asparagine acts as an amino acid exchange factor, thereby contributing to regulation of protein synthesis (9). Given the importance of the anti-leukemia effect mediated by asparaginase-induced asparagine and glutamine depletion, we aimed to determine whether asparaginase exposure has any impact on the plasma levels of other amino acids to potentially discover leukemic cells compensatory responses to asparaginases that may result in resistance to the treatment. To achieve this, we have performed comprehensive plasma amino acid analysis post-asparaginase administration in both AML and PDAC-bearing mouse models and in a clinical trial of patients with R/R AML treated with Erwinaze. To investigate the novelty of our findings, we conducted a systematic literature review of clinical studies that reported treatment with asparaginases to determine the extent of plasma amino acid reporting.

## Methods

### *In vivo* mouse studies

**Figure 1:**
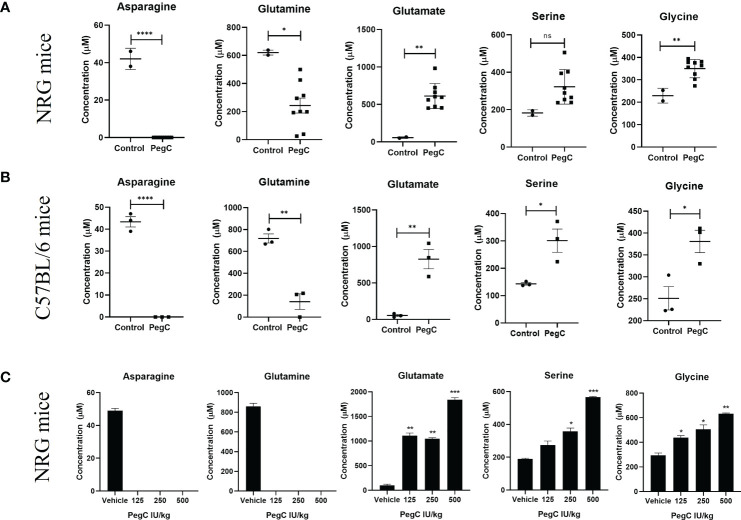
PegC-induced plasma amino acid changes in non-tumor bearing mice. **(A)** NRG mice were treated with vehicle (n=2) or PegC (n=9, 200 IU/kg, IV, 1x/week) for 4 weeks. Plasma was isolated from whole blood obtained at the end of the study and HPLC was performed to measure the concentration of the indicated amino acids. **(B)** C57BL/6 mice were treated with vehicle (n=3) or PegC (n=3, 200 IU/kg, IV, 1x/week) for 4 weeks. Plasma was isolated from whole blood obtained at the end of the study and HPLC was performed to measure the concentration of the indicated amino acids. **(C)** NRG mice were treated with vehicle (n=2) or PegC 125 IU/kg (n=2), PegC 250 IU/kg (n=2), or PegC 500 IU/kg (n=2). Plasma was isolated from whole blood obtained three days after treatment and HPLC was performed to measure the concentration of the indicated amino acids. Statistical analyses were performed using unpaired t- tests. **** p <0.0001, *** p < 0.001, ** p < 0.01, * p <0.05, ns, not significant.

For the tolerability study conducted in immunodeficient NRG (NOD.Cg-*Rag^tm1Mom^IL2rg^tm1Wjl^/SzJ)* mice (**Figure 1A**), mice were treated with either vehicle (n=2) or long-acting Erwinia asparaginase, pegcrisantaspase (PegC). PegC (200 international unit (IU)/kg, intravenously (IV) was administered once weekly (1x/week)) in combination with 5-Fluorouracil (5FU, 10 mg/kg, intraperitoneally (IP), twice weekly (2x/week)) and oxaliplatin (IP, 2x/week) at either 0.5 mg/kg (n=3), 1 mg/kg (n=3), or 5 mg/kg (n=3) for 4 weeks. Mice that received PegC, regardless of concurrent chemotherapy treatment, were grouped together and compared to the vehicle-treated mice. The mice were euthanized 5 days after their last dose of PegC. In the tolerability study in immunocompetent C57BL/6 mice (**Figure 1B**), mice were similarly treated with either vehicle (n=3) or the combination of PegC (200 IU/kg), 5FU (10 mg/kg) and oxaliplatin (5 mg/kg) (n=3) for 4 weeks and plasma was isolated after exsanguination. The mice were euthanized 7 days after their last dose of PegC. Mice that received PegC were grouped together and compared to the vehicle-treated mice. In the non-tumor bearing mouse studies in **Figure 1C**, NRG mice were treated with either vehicle (n=2), or PegC alone at 125 IU/kg (n=2), 250 IU/kg (n=2) or 500 IU/kg (n=2). Mice were euthanized 3 days after treatment and plasma was isolated from whole blood.

**Figure 2:**
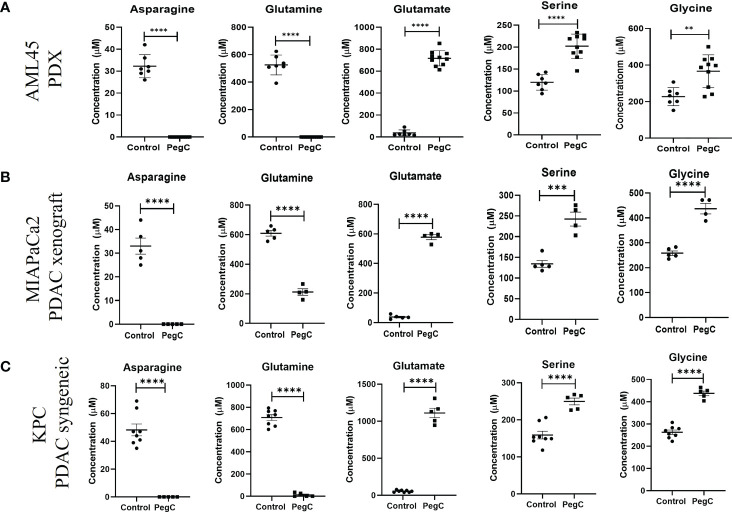
PegC increases serine and glycine in AML PDX and PDAC xenograft/syngeneic models. **(A)** Plasma was isolated from whole blood of AML-bearing NRG mice that did (n=10) or did not (n=7) receive PegC (200 IU/kg, IV, 1x/week) for 4 weeks. HPLC was performed to measure the concentration of the indicated amino acids. **(B)** Plasma was isolated from whole blood of MIAPaCa2 xenograft-bearing NRG mice that did (n=4) or did not (n=5) receive PegC (200IU/kg, IV, 1x/week). HPLC was performed to measure the concentration of the indicated amino acids. **(C)** Plasma was isolated from whole blood of KPC tumor-bearing C57BL/6 mice that did (n=5) or did not (n=8) receive PegC (500 IU/kg, IV, 1x/week). HPLC was performed to measure the concentration of the indicated amino acids. Statistical analyses to compare vehicle and PegC treated groups were performed using unpaired t- tests. **** p < 0.0001, *** p < 0.001, ** p < 0.01.

Establishment of the patient derived xenograft (PDX) AML45-luc model in (**Figure 2A**) has been previously described (10). AML45-luc cells (1 × 10^6^) were injected IV into NRG mice (6-8 weeks old, female). Mice were treated with vehicle, venetoclax (Ven, 75 mg/kg orally (PO), 5x/week), PegC (200 IU/kg, IV, 1x/week) or Ven-PegC. The last dose of PegC was administered on day 36 and mice were euthanized on day 39. Plasma was isolated from whole blood after exsanguination. Since no significant differences in amino acid levels were noted in Ven-treated mice, all mice that received PegC (PegC alone and Ven+PegC) were grouped together (n=10) and compared to mice that did not receive PegC (vehicle and Ven alone) (n=7).

For the MIAPaCa2 PDAC xenograft model (**Figure 2B**), MIAPaCa2 cells (3 x 10^6^) were injected subcutaneously into the flank of female NRG mice. When tumors were palpable (150mm^3^), mice were treated with either vehicle or PegC at a dose of 200 IU/kg IV once a week. For the KPC syngeneic pancreatic adenocarcinoma model in **Figure 2C**, KPC cells (3 x 10^6^) were injected subcutaneously into the flank of male or female C57BL/6 mice. When tumors were palpable (150mm^3^), mice were treated with either vehicle or PegC at a dose of 200 IU/kg IV once a week. Plasma was isolated from whole blood after exsanguination. PegC was provided by Jazz Pharmaceuticals.

### Western Blot Analysis

Tumors were lysed using tissue extraction reagent (ThermoFisher Scientific, Waltham, MA) supplemented with protease and phosphatase inhibitor cocktails (Sigma Aldrich). Lysates were incubated on ice for 10min then centrifuged at 10,000 g at 4°C for 15 min. Protein content of lysates was determined and lysates were separated on 4-15% polyacrylamide gels (BioRad, Hercules,CA), then transferred onto polyvinylidene difluoride (PVDF) membranes (BioRad). Membranes were blocked with 5% non-fat milk in tris-buffered saline with 0.1% Tween 20 (TBST), incubated with primary antibodies at 4°C overnight, then incubated with HRP-conjugated secondary antibody for 1h. Bands were visualized using Clarity Western Enhanced Chemiluminescence (ECL)substrate (BioRad). Densitometric analyses were performed using ImageJ (NIH).

### Clinical study with short-acting crisantaspase

Adult patients (n=5) with R/R AML following at least one line of therapy were eligible for the study. Crisantaspase (Erwinaze) was administered at a dose of 25,000 IU/m^2^ three days a week (Monday, Wednesday, Friday) for a total of 6 doses over 2 weeks. No concurrent anti-neoplastic agent was permitted. Plasma amino acid levels were measured pre-study and subsequently before administration of Erwinaze on Wednesday and Friday (approximate 48 hours trough) and on Monday morning of the 2^nd^ week (approximate 72 hours trough). Erwinaze was provided by Jazz Pharmaceuticals. As of July 23, 2021, Erwinaze is no longer commercially available in the US.

### Plasma amino acid measurement

For mouse studies, plasma was isolated from whole blood and delivered on ice to the University of Maryland Pathology Associates Biochemical Genetics Laboratory for plasma quantitative amino acid analysis. For human clinical samples, 5-8 mL blood was collected in a sodium/lithium heparin tube and kept on ice. Within 40 minutes of collection, the plasma was separated by centrifugation (1100g × 5 minutes at 4°C) and frozen at -20°C until analysis. Free amino acid concentrations were measured using a Biochrom 30 or Biochrom 30+ Amino Acid Analyzer (Biochrom Ltd., Cambridge, UK) by cation-exchange chromatography and ninhydrin detection according to the manufacturer’s instructions. Results were quantified using commercially available calibration standards and normalized to the internal standard, s-2-aminoethylcysteine and reported as micromole per liter (µM). Two quality control standards were evaluated as an unknown at the beginning of each set of sample runs.

### Statistics

To compare plasma amino acid concentrations between the two groups of mice that did or did not receive PegC, unpaired t-tests were performed using GraphPad Prism 8. For clinical samples, unpaired t-tests were used to compare changes from baseline levels over time.

### Literature review

A literature review was undertaken to determine available clinical data on serum and/or plasma amino acid concentrations after asparaginase therapy for patients with any malignancy. On July 15, 2021, a PubMed search of clinical and randomized clinical trials over the last 41 years was completed in accordance with the search strategy detailed in **Figure 4**. After the initial search, articles’ full-texts were screened for inclusion for further analysis. To be included, full-text articles had to be in English and report prospective clinical data from human subjects, the use of asparaginase therapy in subjects with a malignancy, and serum/plasma amino acid concentrations at specific time points after treatment initiation. Articles were excluded if they reported data from an already included study. Included studies were then analyzed for age groups of participants, types of malignancy studied, type of asparaginase utilized, and serum/plasma amino acid concentrations measured.

## Results

### Crisantaspase increases plasma levels of serine and glycine in both non-tumor bearing and tumor-bearing mice

To examine the impact of crisantaspase treatment on the plasma levels of all amino acids, we measured the concentrations of amino acids in the plasma of both immunodeficient NRG and immunocompetent C57BL/6 mice treated with PegC. Mice were treated with PegC for 4 weeks and plasma was processed from whole blood obtained at the end of the study. For NRG mice, outside of the expected decreases in asparagine and glutamine and increases in aspartate and glutamate, mean levels of glycine increased from 230 ± 33 µM to 350 ± 41 µM (p=0.0038) and though not statistically significant, a notable increase in plasma serine from 182 ± 17 µM to 322 ± 92 µM was also observed (**Figure 1A**; **Supplemental Table 1**). C57BL/6 mice exposed to PegC had significant increases in plasma serine from 143 ± 8 µM to 301± 74 µM (p=0.02) and glycine from 251 ± 46 µM to 381 ± 44 µM (p=0.02) (**Figure 1B**; **Supplemental Table 2**). Other amino acids significantly increased by PegC in both NRG and C57BL/6 mice include threonine and α-amino-n-butyric acid (**Supplemental Tables 1, 2**).

To confirm the increase in plasma serine and glycine induced by PegC, we treated NRG mice once with an increasing dose of PegC (125, 250, and 500 IU/kg) alone and euthanized mice three days after treatment. Both serine and glycine were increased by PegC in a dose dependent manner (**Figure 1C**). Serine significantly increased from 190 ± 6 µM to 357 ± 29 µM (p=0.01) in the 250 IU/kg group and 565 ± 8 µM (p=0.0004) in the 500 IU/kg group. Glycine also significantly increased from 295 ± 25 µM to 438 ± 24 µM (p=0.02) in the 125 IU/kg group, 507 ± 50 µM (p=0.03) in the 250 IU/kg group, and 633 ± 8 µM (p=0.003) in the 500 IU/kg group.

To establish the impact of PegC on plasma amino acid levels in a tumor-bearing mouse model, we measured amino acids obtained from our recent study using a patient derived xenograft model of complex karyotype AML (10). We compared comprehensive plasma amino acid concentrations between mice that did (n=10) and did not (n=7) receive PegC. As previously published, mice treated with PegC had undetectable levels of glutamine and asparagine and glutamate levels significantly increased (10). Importantly, consistent with our observation in non-tumor bearing mice, PegC also significantly increased plasma levels of serine, from 120 ± 18 µM to 202 ± 28 µM (p<0.0001) and glycine, from 227 ± 50 µM to 366 ± 90 µM (p=0.002) (**Figure 2A**; **Supplemental Table 3**). In line with our findings in non-tumor bearing models, PegC also significantly increased the levels of threonine (p<0.0001) and α-amino-n-butyric acid (p=0.006) (**Supplemental Table 3**).

To extend our investigation into a solid tumor model, we measured plasma amino acids in NRG mice bearing PDAC xenografts (MIAPaCa2) that were vehicle treated (n=5) or treated with PegC (n=4). PegC treatment significantly increased plasma levels of serine, from 134 ± 18 µM to 242 ± 34 µM (p=0.0004) and glycine, from 258 ± 20 µM to 437 ± 42 µM (p<0.0001) (**Figure 2B**; **Supplemental Table 4**). To examine the impact of PegC in an immunocompetent tumor model, we then measured plasma amino acids in C57BL/6 mice bearing KPC syngeneic PDAC tumors that were vehicle treated (n=8) or treated with PegC (n=5), Once again, we observed that PegC treatment significantly increased plasma levels of serine, from 159 ± 30 µM to 250 ± 20 µM (p=0.0001) and glycine, from 263 ± 27 µM to 438 ± 23 µM (p <0.000001) (**Figure 2C**; **Supplemental Table 5**).

### Crisantaspase treatment upregulates serine biosynthesis enzymes in PDAC tumors

**Figure 3:**
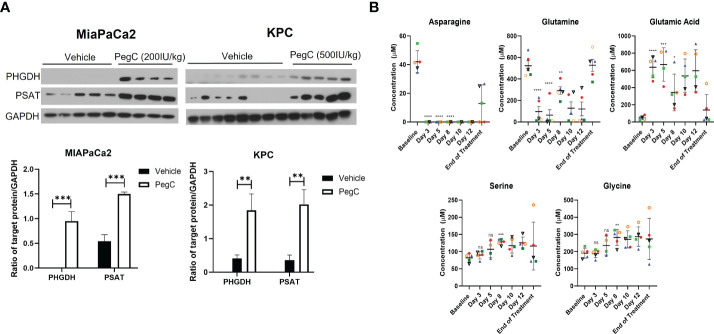
PegC upregulates serine biosynthesis enzymes in PDAC tumors **(A)** Western blot analysis of MIAPaCa2 and KPC tumor lysates from mice either vehicle treated or treated with the indicated dose of PegC **(B)** Plasma amino acid levels from Erwinaze clinical trial. Changes in plasma amino acid levels following Erwinaze treatment. Each marker represents a different patient (n=5). Statistical analyses were performed using unpaired t- tests. **** p < 0.0001, *** p < 0.001, **p < 0.01, *p <0.05, ns, not significant.

Since PegC treatment increased plasma levels of serine in both healthy and tumor-bearing mice, we wanted to determine the impact of PegC on serine biosynthesis in tumor tissue. Tumor lysates from the MIAPaCa2 and KPC studies in **Figure 2** were immunoblotted for phosphoglycerate dehydrogenase (PHGDH) and phosphoserine aminotransferase (PSAT), critical enzymes for *de novo* serine biosynthesis. As shown in **Figure 3A**, PegC treatment resulted in a significant upregulation of PHGDH and PSAT in both MIAPaCa2 and KPC tumors.

### Erwinaze clinical trial data confirms crisantaspase-induced increases in plasma serine and glycine

To further investigate the effect of crisantaspase on plasma amino acid levels, we examined amino acid concentrations measured at different time points in 5 patients with R/R AML treated with Erwinaze over the course of a clinical trial (4). Asparagine became undetectable in the plasma of all 5 patients after the first dose of Erwinaze after measurement on day 3 of the study and remained undetectable for the entire two weeks of active treatment (**Figure 3B**). In 3 of the 5 patients, glutamine was reduced to below 20 µM after only one dose of Erwinaze. By day 8 of the study, there was a significant increase over baseline in the average level of serine, from 82 ± 11 µM to 128 ± 8 µM (p=0.0007) and glycine, from 191 ± 26 µM to 282 ± 467 µM (p=0.006) (**Figure 3B**). Erwinaze treatment resulted in significant alterations of several amino acids between baseline and day 8 of the study, and consistent with our *in vivo* findings, levels of threonine (p=0.001) and α-amino-n-butyric acid (p=0.03) were significantly increased (**Supplemental Table 6**).

### Clinical studies with asparaginase treatment report limited amino acid panel analysis

**Figure 4:**
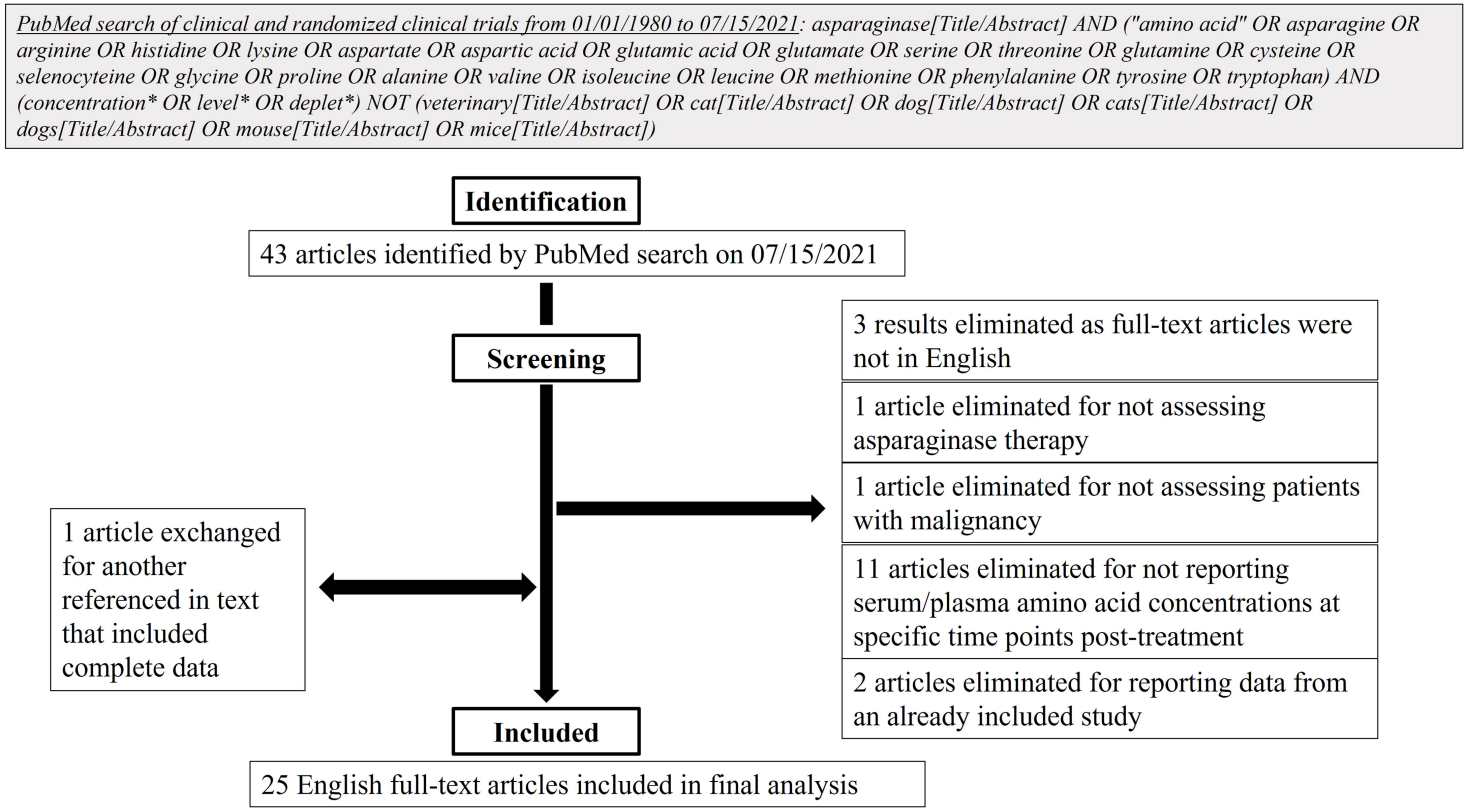
Flow diagram of literature search design and methods.

After identification of 43 articles from the initial PubMed search, 25 were included for further analysis based on the screening strategy (**Figure 4**). Of these articles, the majority reported serum/plasma amino acid levels after asparaginase treatment initiation in the pediatric population (age <18 years, n=17 articles). Four articles included both adults and children and four reported exclusively on adults (age ≥18 years). Of the articles including adults, only four incorporated subjects ≥31 years and two incorporated participants ≥56 years. These articles primarily analyzed asparaginase therapy in ALL (n=23) with a smaller number reporting on AML (n=3), chronic myeloid leukemia in blast phase (n=1), and non-Hodgkin’s lymphoma (n=1). One study analyzed asparaginase therapy in participants with a conglomerate of primarily solid tumors: melanoma, small cell lung cancer, non-small cell lung cancer, sarcoma, colon cancer, cholangiocarcinoma, renal cell carcinoma, bladder cancer, and malignancy with unknown primary. The included studies commonly reported on the use of native *E. coli* asparaginase (n=12) or one of its derivatives, pegylated *E. coli* asparaginase with the standard succinimidyl succinate linker (n=9); *E. coli* L-asparaginase encapsulated within erythrocytes (n=2); or *E. coli* calaspargase pegol, pegylated with a succinimidyl carbonate linker (n=1). Other studies detailed the effects of asparaginases from alternative sources: the *Erwinia chrysanthemi* enzyme (n=9), recombinant asparaginase (n=3), or succinylated *Acinetobacter* glutaminase-asparaginase (n=1). The plasma/serum concentrations of only asparagine (n=24 studies), glutamine (n=10), glutamic acid (n=10), and aspartic acid (n=6) were reported in the included articles, and only two articles reported on the levels of other amino acids in non-plasma/serum spaces, specifically the cerebrospinal spinal fluid or intracellularly in leukemic cells. A list of the included studies and the participant groups and variables analyzed by each are included in **Supplementary Table 7**.

## Discussion

Here we report that in comprehensive plasma amino acid analysis following exposure to crisantaspase, we observed a reproducible crisantaspase-induced increase in plasma serine and glycine in both mouse and human plasma samples. Systematic literature review of clinical studies with asparaginases revealed that only levels of glutamine/glutamate and asparaginase/aspartate have been published post-asparaginase administration, highlighting the novelty of our findings.

It is plausible that restriction of circulating asparagine and glutamine induced by crisantaspase (and other asparaginases) could induce upregulation of *de novo* amino acid biosynthesis to compensate for reduced availability. In mammalian cells, amino acid starvation triggers initiation of the amino acid response (AAR). Decreased intracellular amino acids results in an accumulation of unloaded tRNAs, which can activate general control nonderepressible 2 (GCN2). GCN2 then phosphorylates the eukaryotic initiation factor 2a (eIF2a), causing a shift from cap-dependent translation to cap-independent translation of transcripts that harbor an upstream open reading frames (such as activating transcription factor 4 [ATF4]) or an internal ribosome entry site, such as c-Myc. Translation of the transcription factors ATF4 and c-Myc leads to the expression of genes involved in amino acid biosynthesis and membrane transport (11–13).

Of note, ATF4 and c-Myc promote transcription of PHGDH and PSAT, enzymes involved in *de novo* serine synthesis. Serine is a non-essential amino acid important for the generation of nucleic acid and lipid precursors. Serine is also a substrate in the synthesis of the amino acid glycine, an important carbon-donor for the initiation of the folate cycle (14, 15). As one of the three amino acids that comprise the powerful antioxidant glutathione (GSH), glycine also contributes to maintaining cellular redox homeostasis. It can be speculated - and further research is ongoing - that the increase in plasma serine and glycine levels in response to crisantaspase is due to the initiation of the AAR pathway, which increases serine biosynthesis and subsequent conversion of serine to glycine.

Our findings offer insights into potential resistance mechanisms to asparaginase therapy. Serine metabolism has an emerging role in oncogenesis and as a therapeutic target. Tumors have been shown to increase expression of enzymes involved in serine biosynthesis to reduce dependency on exogenous supply. Several solid tumors, including triple negative breast cancer, lung adenocarcinoma, and pancreatic cancer overexpress PHGDH (16–18), and inhibition of PHGDH can suppress cancer cell proliferation both *in vitro* and in PDX models (19, 20). In AML, high PHGDH expression has been reported as a negative prognostic marker (21), and downregulation of serine biosynthesis pathway enzymes results in decreased GSH and increased reactive oxygen species production (22, 23). In response to glutamine withdrawal, leukemia cells upregulate PHGDH and PSAT, suggesting that targeting both serine and glutamine metabolism may result in synergistic anti-leukemic activity (24). Indeed, Polet et al. demonstrated that reducing serine supply through PHGDH gene silencing complements perturbation of glutamine metabolism to inhibit leukemia cell growth (24). In pancreatic cancer, PHGDH enhances mRNA translation by interacting with eIF4A1 and eIF4E, thereby promoting cancer development (25). Although the increase in plasma serine observed after asparaginase is not necessarily a neoplastic-dependent phenomenon, cancer cells may be able to take advantage of increased serine to fuel cell proliferation. Targeting serine metabolism in combination with glutamine depletion by asparaginases may be a promising therapeutic option for both solid and hematologic neoplasms.

In addition to serine and glycine, we observed an increase in levels of α-amino-n-butyric acid (ABA) and threonine following crisantaspase treatment in our models. Threonine is an essential amino acid that is supplied through dietary intake. The mechanism by which crisantaspase increases plasma threonine is not clear; however, since asparagine has been established as an amino acid exchange factor for threonine (9), a potential explanation may be that asparagine depletion decreases threonine transport into the cell resulting in accumulation in the plasma. ABA is not involved in protein synthesis and is generally considered a non-specific marker of increased protein catabolism (26). ABA is a product of the metabolism of serine, glycine, methionine, and threonine (27, 28), and we posit that the increase observed in plasma can be attributed to the increase in serine and glycine initiated by the AAR.

Outside of our literature review, we found one study that measured seven plasma amino acids in pediatric ALL patients that received asparaginase-containing chemotherapy and contrary to our findings, they reported decreased levels of serine and threonine (29). Asparaginase was administered alongside several other anti-neoplastic agents in this study, therefore the toxicity of the intensive chemotherapy regimen may have contributed to the observed decrease in plasma amino acids. Furthermore, the asparaginases used in the study were *E. coli*-derived, whereas our plasma amino acid data were generated from studies using crisantaspases, which have a higher glutaminase activity (1, 2). It would be interesting to further investigate how *E. coli* and *Erwinia* asparaginases may differentially impact plasma amino acids.

## Conclusions

To the best of our knowledge, this is the first time that full plasma amino acid profiles have been reported for mice and humans treated with an asparaginase. By analyzing the full amino acid panel, rather than only glutamine and asparagine, we were able to see significant changes in serine and glycine levels, which may be an indication of potential mechanisms of asparaginase resistance or provide promising new therapeutic targets. Ongoing studies are focused on exploring the mechanism of asparaginase-induced increase in serine as well as dual targeting of glutamine and serine metabolic pathways in AML. Preclinically, we are investigating the effect of an *E.coli*-derived asparaginase on plasma amino acid levels. Clinically, the full panel of plasma amino acid levels are being measured in two ongoing clinical trials testing PegC in R/R AML (NCT04666649) and calasparagase pegol-mknl in patients with newly diagnosed AML (NCT04953780).

## Data availability statement

The original contributions presented in the study are included in the article/**Supplementary Material**. Further inquiries can be directed to the corresponding author.

## Ethics statement

The studies involving human participants were reviewed and approved by University of Maryland Baltimore Institutional Review Board. The patients/participants provided their written informed consent to participate in this study. The animal study was reviewed and approved by University of Maryland Baltimore Institutional Animal Care and Use Committee. All procedures performed in studies involving human participants were in accordance with the ethical standards of the institutional and/or national research committee and with the 1964 Helsinki declaration and its later amendments or comparable ethical standards. For all animal studies, mice were housed under pathogen-free conditions in a University of Maryland Baltimore Association for Assessment and Accreditation of Laboratory Animal Care (AAALAC)- accredited facility. All experiments were conducted in compliance with Public Health Service (PHS) guidelines for animal research.

## Author contributions

AE and DB conceived the idea and designed the study. JC conducted the literature review. ES performed plasma amino acid analysis. KM and DB analyzed the amino acid data and designed figures. DB performed tumor immunoblotting. BC-C processed clinical plasma samples. XM, KT, and RL carried out *in vivo* experiments under Institutional Animal Care and Use Committees (IACUC) protocols. DB and JC wrote the first draft of the manuscript. All authors contributed to the article and approved the submitted version.

## Funding

This study was supported by a research grant provided by Jazz Pharmaceuticals to AE. This work was partially supported by the University of Maryland Greenebaum Comprehensive Cancer Center Support grant (P30CA134274) and the State of Maryland’s Cigarette Restitution Funds.

## Conflict of interest

AE has received research grants from Jazz Pharmaceuticals, Amgen, NewLinks, and Servier. AE is a global oncology advisory board member for Amgen and has served as an advisory board member for Genentech, Servier, Kite Pharma, and Secura Bio. AE and RL are Co-Founders and Scientific Advisors for KinaRx, Inc.

The remaining authors declare that the research was conducted in the absence of any commercial or financial relationships that could be construed as a potential conflict of interest.

## Publisher’s note

All claims expressed in this article are solely those of the authors and do not necessarily represent those of their affiliated organizations, or those of the publisher, the editors and the reviewers. Any product that may be evaluated in this article, or claim that may be made by its manufacturer, is not guaranteed or endorsed by the publisher.

## Abbreviations

AAR, amino acid response; ABA, Alpha-amino-n-butyric acid; ALL, Acute lymphoblastic leukemia; AML, Acute myeloid leukemia; AMMol, acute myelomonocytic leukemia; ASNS, asparagine synthetase; ATF4, activating transcription factor 4; CML, chronic myeloid leukemia; eIF2a, eukaryotic initiation factor 2a; GCN2, general control nonderepressible 2; GSH, glutathione; NEAA, non-essential amino acids; NRG, NOD.Cg-*Rag^tm1Mom^IL2rg^tm1Wjl^/SzJ;* PDAC, pancreatic ductal adenocarcinoma; PDX, patient-derived xenograft; PegC, pegcrisantaspase; PHGDH, phosphoglycerate dehydrogenase; PSAT, phosphoserine aminotransferase; R/R, relapsed/refractory.
