# Supplementary material for: Erwinia asparaginase (crisantaspase) increases plasma levels of serine and glycine
Source: Front Oncol. 2022 Dec 12;12:1035537. doi: 10.3389/fonc.2022.1035537 (PMC9790920; doi:10.3389/fonc.2022.1035537)
Supplement: Supplementary file 1 [file DataSheet_1.pdf]

Non-tumor bearing NRG mice

	Concentration (μM) in mice that did not receive PegC (n=2)	Concentration (μM) in mice that received PegC (n=9)	p-value
**Asparagine**	42.0	0.0	**<0.000001**
**Glutamine**	621.0	243.0	**0.0106**
**Glutamate**	57.0	612.0	**0.0015**
**Histidine**	102.0	103.0	0.9064
**Glycine**	230.0	350.0	**0.0038**
**Threonine**	213.0	296.0	**0.0166**
**Serine**	182.0	322.0	0.0702
**Citrulline**	122.0	99.0	0.1201
**α-Amino-n-Butyric Acid**	3.0	7.0	**0.0260**
**Valine**	302.0	301.0	0.9847
**1-Methylhistidine**	6.0	8.0	**0.0403**
**Phosphoethanolamine**	9.0	9.0	0.8745
**Aspartate**	25.0	55.0	**0.0375**
**Sarcosine**	0.0	0.0	--
**α-Aminiadipic Acid**	9.0	9.0	0.8174
**Proline**	149.0	180.0	0.5176
**Taurine**	591.0	469.0	0.2138
**Alanine**	576.0	648.0	0.6671
**Phosphoserine**	15.0	17.0	0.4304
**Cysteine**	7.0	13.0	0.1516
**Methionine**	115.0	102.0	0.7484
**Cystathionine**	0.0	0.0	
**Isoleucine**	119.0	125.0	0.6800
**Leucine**	243.0	238.0	0.8844
**Tyrosine**	101.0	103.0	0.9212
**Phenylalanine**	104.0	101.0	0.8605
**Homocysteine**	5.00	2.0	0.2230
**Ethanolamine**	0.0	0.0	
**Ornithine**	292.0	236.0	0.5239
**Lysine**	414.0	492.0	0.2446
**Tryptophan**	107.0	97	0.4365
**Arginine**	100.0	203.0	0.2266
**Anserine**	0.0	0.0	--
**Carnosine**	0.0	0.0	--
**Hydroxyproline**	0.0	0.0	--
**Hydroxylysine**	0.0	0.0	--
**B-Aminoisobutyric Acid**	0.0	0.0	--
**Gaba-Aminobutyric Acid**	0.0	0.0	--
**Beta-alanine**	0.0	0.0	--
