# Supplementary material for: Erwinia asparaginase (crisantaspase) increases plasma levels of serine and glycine
Source: Front Oncol. 2022 Dec 12;12:1035537. doi: 10.3389/fonc.2022.1035537 (PMC9790920; doi:10.3389/fonc.2022.1035537)
Supplement: Supplementary file 2 [file DataSheet_2.pdf]

Non-tumor bearing C57BL/6 mice

	Concentration (μM) in mice that did not receive PegC (n=3)	Concentration (μM) in mice that received PegC (n=3)	p-value
**Asparagine**	43.0	0.0	**0.000049**
**Glutamine**	719.0	142.0	**0.00219**
**Glutamate**	54.0	826.0	**0.0043**
**Histidine**	75.0	85.0	0.1892
**Glycine**	251.0	381.0	**0.0243**
**Threonine**	163.0	307.0	**0.0174**
**Serine**	143.0	301.0	**0.021**
**Citrulline**	90.0	84.0	0.308
**α-Amino-n-Butyric Acid**	15.0	23.0	**0.0022**
**Valine**	230.0	229.0	0.957
**1-Methylhistidine**	16.0	9.0	**0.00318**
**Phosphoethanolamine**	18.0	10.0	0.1078
**Aspartate**	30.0	49.0	0.1053
**Sarcosine**	0.0	0.0	--
**A-Aminiadipic Acid**	0.0	0.0	--
**Proline**	99.0	192.0	0.1253
**Taurine**	689.0	419.0	0.1224
**Alanine**	414.0	1192.0	**0.0282**
**Phosphoserine**	20.0	13.0	0.0587
**Cysteine**	11.0	12.0	0.5406
**Methionine**	73.0	89.0	0.4446
**Cystathionine**	0.0	0.0	--
**Isoleucine**	102.0	90.0	0.2989
**Leucine**	182.0	197.0	0.547
**Tyrosine**	85.0	92.0	0.7769
**Phenylalanine**	74.0	76.0	0.8796
**Homocysteine**	0.00	0.0	--
**Ethanolamine**	0.0	0.0	--
**Ornithine**	109.0	164.0	0.1376
**Lysine**	354.0	531.0	0.0234
**Tryptophan**	68.0	77	0.7137
**Arginine**	115.0	118.0	0.9391
**Anserine**	0.0	0.0	--
**Carnosine**	0.0	0.0	--
**Hydroxyproline**	0.0	0.0	--
**Hydroxylysine**	0.0	0.0	--
**B-Aminoisobutyric Acid**	0.0	0.0	--
**Gaba-Aminobutyric Acid**	0.0	0.0	--
**Beta-alanine**	0.0	0.0	--
