# Supplementary material for: Erwinia asparaginase (crisantaspase) increases plasma levels of serine and glycine
Source: Front Oncol. 2022 Dec 12;12:1035537. doi: 10.3389/fonc.2022.1035537 (PMC9790920; doi:10.3389/fonc.2022.1035537)
Supplement: Supplementary file 3 [file DataSheet_3.pdf]

AML45-luc tumor bearing mice

	Concentration (μM) in mice that did not receive PegC (n=7)	Concentration (μM) in mice that received PegC (n=10)	p-value
**Asparagine**	32.29	0	**<0.000001**
**Glutamine**	525.4	0	**<0.000001**
**Glutamate**	37.57	717.3	**<0.000001**
**Histidine**	77.71	67.3	**0.016977**
**Glycine**	227.1	366	**0.002132**
**Threonine**	116.1	192.7	**0.000055**
**Serine**	119.9	202.1	**0.000005**
**Citrulline**	135.4	175.9	0.071657
**A-Amino-n-Butyric Acid**	3.286	13.7	**0.005944**
**Valine**	208.1	245.8	0.028213
**1-Methylhistidine**	7	8.8	**0.008207**
**Phosphoethanolamine**	282.9	483.6	0.088491
**Aspartate**	26.57	35.7	0.172
**Sarcosine**	15.29	0	0.080679
**A-Aminiadipic Acid**	0	0.8	0.420421
**Proline**	94	93.3	0.930416
**Taurine**	337.3	346.9	0.861642
**Alanine**	366	416.2	0.140677
**Phosphoserine**	17.86	26.3	0.172835
**Cysteine**	12.57	8.6	0.146814
**Methionine**	54.29	49.2	0.594126
**Cystathionine**	0	0.2	0.420421
**Isoleucine**	92.71	92.9	0.971848
**Leucine**	157.3	166.8	0.348324
**Tyrosine**	68.43	49.1	**0.000638**
**Phenylalanine**	73.86	69.7	0.2535
**Homocysteine**	1.143	1	0.788236
**Ethanolamine**	0	1.7	0.234043
**Ornithine**	119.3	134.9	0.547635
**Lysine**	267.7	310.2	0.148207
**Tryptophan**	90.86	100	0.233692
**Arginine**	67.29	107.5	0.109302
**Anserine**	0	0	--
**Carnosine**	0	0	--
**Hydroxyproline**	0	0	--
**Hydroxylysine**	0	0	--
**B-Aminoisobutyric Acid**	0	0	--
**Gaba-Aminobutyric Acid**	0	0	--
**Beta-alanine**	0	0	--
