# Supplementary material for: Erwinia asparaginase (crisantaspase) increases plasma levels of serine and glycine
Source: Front Oncol. 2022 Dec 12;12:1035537. doi: 10.3389/fonc.2022.1035537 (PMC9790920; doi:10.3389/fonc.2022.1035537)
Supplement: Supplementary file 4 [file DataSheet_4.pdf]

MiaPaca2 xenograft mice

	Concentration (µM) in mice that did not receive PegC (n=5)	Concentration (µM) in mice that received PegC (n=4)	p-value
**Asparagine**	33.0	0.0	**<0.0001**
**Glutamine**	609.2	212.5	**<0.00001**
**Glutamate**	37.4	577.8	**<0.000001**
**Histidine**	73.4	70.8	0.5265
**Glycine**	258.4	437.0	**<0.0001**
**Threonine**	114.2	194.8	**0.0031**
**Serine**	134.2	242.5	**0.0004**
**Citrulline**	124.4	122.8	0.8700
**A-Amino-n-Butyric Acid**	4.8	6.3	0.3277
**Valine**	203.2	236.3	0.1107
**Methylhistidine**	10.8	14.8	**0.0075**
**Phosphoethanolamine**	385.6	266.8	0.1695
**Aspartate**	18.2	36.3	0.0037
**Sarcosine**	0.0	0.0	--
**A-Aminiadipic Acid**	0.0	0.0	--
**Proline**	99.2	105.5	0.5546
**Taurine**	474.4	395.3	0.3052
**Alanine**	364.8	471.8	0.0501
**Phosphoserine**	27.4	17.3	**0.0209**
**Cysteine**	5.8	6.5	0.4070
**Methionine**	52.8	50.3	0.8104
**Cystathionine**	2.2	3.0	0.2655
**Isoleucine**	89.4	93.0	0.5678
**Leucine**	158.8	174.8	0.3687
**Tyrosine**	69.0	52.8	**0.0067**

**Phenylalanine**	74.8	76.8	0.6660
**Homocysteine**	0.00	0.8	0.2923
**Ethanolamine**	4.6	0.0	0.0747
**Ornithine**	149.0	119.3	0.3441
**Lysine**	285.8	319.0	0.0861
**Tryptophan**	62.4	80	0.1130
**Arginine**	40.0	127.5	0.0033
**Anserine**	0.0	0.0	--
**Carnosine**	0.0	0.0	--
**Hydroxyproline**	0.0	0.0	--
**Hydroxylysine**	0.0	0.0	--
**B-Aminoisobutyric Acid**	0.0	0.0	--
**Gaba-Aminobutyric Acid**	0.0	0.0	--
**Beta-alanine**	0.0	0.0	--
