# Supplementary material for: Erwinia asparaginase (crisantaspase) increases plasma levels of serine and glycine
Source: Front Oncol. 2022 Dec 12;12:1035537. doi: 10.3389/fonc.2022.1035537 (PMC9790920; doi:10.3389/fonc.2022.1035537)
Supplement: Supplementary file 5 [file DataSheet_5.pdf]

KPC syngeneic model

	Concentration (µM) in mice that did not receive PegC (n=8)	Concentration (µM) in mice that received PegC (n=5)	p-value
**Asparagine**	48.0	0.0	**<0.00001**
**Glutamine**	708.0	12.0	**<0.000001**
**Glutamate**	55.0	1110.6	**<0.000001**
**Histidine**	75.0	82.4	0.1330
**Glycine**	263.0	437.8	**<0.000001**
**Threonine**	162.0	277.0	**<0.00001**
**Serine**	159.0	249.6	**0.0001**
**Citrulline**	64.0	91.4	**0.0010**
**A-Amino-n-Butyric Acid**	7.0	10.4	0.0450
**Valine**	155.0	196.2	**0.0060**
**Methylhistidine**	16.0	18.4	**0.0178**
**Phosphoethanolamine**	9.0	8.8	0.9480
**Aspartate**	21.0	42.8	**0.0060**
**Sarcosine**	0.0	0.0	--
**A-Aminiadipic Acid**	0.0	0.0	--
**Proline**	108.0	107.6	0.9940
**Taurine**	539.0	517.8	0.8100
**Alanine**	536.0	784.2	**0.0005**
**Phosphoserine**	4.0	4.6	0.7760
**Cysteine**	7.0	7.0	0.6760
**Methionine**	89.0	67.0	0.0751
**Cystathionine**	0.0	0.0	--
**Isoleucine**	74.0	90.0	**0.0102**
**Leucine**	147.0	159.0	0.2780
**Tyrosine**	66.0	60.2	0.2500

**Phenylalanine**	74.0	63.6	0.0720
**Homocysteine**	0.00	0.0	
**Ethanolamine**	0.0	0.0	
**Ornithine**	99.0	152.0	0.1390
**Lysine**	384.0	411.0	0.5110
**Tryptophan**	89.0	93	0.5790
**Arginine**	113.0	97.6	0.5510
**Anserine**	0.0	0.0	--
**Carnosine**	0.0	0.0	--
**Hydroxyproline**	0.0	0.0	--
**Hydroxylysine**	0.0	0.0	--
**B-Aminoisobutyric Acid**	0.0	0.0	--
**Gaba-Aminobutyric Acid**	0.0	0.0	--
**Beta-alanine**	0.0	0.0	--
