# Supplementary material for: Erwinia asparaginase (crisantaspase) increases plasma levels of serine and glycine
Source: Front Oncol. 2022 Dec 12;12:1035537. doi: 10.3389/fonc.2022.1035537 (PMC9790920; doi:10.3389/fonc.2022.1035537)
Supplement: Supplementary file 6 [file DataSheet_6.pdf]

Black triangle

Amino Acid	Baseline	Day 3	Day 8	Day 10	Day 12	EOT
Phosphoserine	0	0	5	5	2	0
Taurine	66	81	60	70	53	48
Phosphoethanolamine	0	0	0	0	0	3
Aspartic Acid	6	15	16	29	22	21
Hydroxyproline	0	0	0	0	0	0
Threonine	73	100	212	277	385	111
Serine	64	98	116	143	153	83
Asparagine	37.6	0.0	0.0	0.0	0.0	25.0
Glutamic Acid	51.3	754.3	201.0	385.4	406.9	40.4
Glutamine	487.9	27.6	278.5	277.5	318.1	562.7
Sarcosine	0	0	0	0	0	0
A-Aminiadipic Acid	14	0	4	13	0	0
Proline	151	204	204	242	273	195
Glycine	157	181	211	275	300	263
Alanine	267	323	263	324	448	339
Citrulline	37	32	25	31	31	24
A-Amino-n-Butyric Acid	19	26	36	33	30	21
Valine	270	471	353	393	398	256
Cystine	51	60	63	66	62	39
Methionine	13	18	28	44	47	19
Cystathionine	0	0	4	0	0	0
Isoleucine	65	114	98	119	128	47
Leucine	137	233	195	232	226	109
Tyrosine	39	71	94	122	131	74
Beta-alanine	0	0	0	0	0	0
Phenylalanine	47	79	145	124	132	86
B-Aminoisobutyric Acid	13	0	0	0	0	0
Homocystine	0	0	0	0	0	0
G-Aminobutyric Acid	0	0	0	0	0	0
Ethanolamine	50	64	0	0	0	0
Hydroxylysine	0	0	0	0	0	0
Ornithine	96	97	95	126	114	75
Lysine	173	249	228	320	343	192
1-Methylhistidine	4	12	3	9	17	5
Histidine	81	102	75	83	95	93
Tryptophan	60	68	65	71	77	58
3-Methylhistidine	6	11	7	8	9	8
Anserine	0	0	0	0	0	0
Carnosine	0	0	0	0	0	0
Arginine	76	55	59	91	101	77

Blue triangle

Amino Acid	Baseline	Day 3	Day 5	Day 8	Day 10	Day 12	EOT
Phosphoserine	0	0	5	2	2	2	0
Taurine	52	71	67	68	65	77	36
Phosphoethanolamine	0	0	0	0	0	0	0
Aspartic Acid	0	14	16	16	13	14	28
Hydroxyproline	0	0	0	0	0	0	0
Threonine	121	153	134	316	240	221	108
Serine	75	101	95	136	114	109	72
Asparagine	54.7	0	0	0	0	0	13
Glutamic Acid	19.9	536	624	318	527	527	26
Glutamine	670.7	227	27	308	85	28	563
Sarcosine	0	0	0	0	0	0	0
A-Aminiadipic Acid	10	13	7	9	9	10	0
Proline	219	215	170	229	191	211	223
Glycine	231	212	202	325	287	272	228
Alanine	424	362	301	491	379	437	313
Citrulline	46	46	33	42	36	35	40
A-Amino-n-Butyric Acid	15	36	25	25	31	26	17
Valine	176	328	278	283	281	253	155
Cystine	58	63	66	66	64	45	53
Methionine	14	19	22	32	20	22	11
Cystathionine	0	0	0	0	0	0	0
Isoleucine	47	69	67	68	52	53	29
Leucine	79	173	168	154	144	142	72
Tyrosine	58	56	71	88	58	63	57
Beta-alanine	0	0	0	0	0	0	0
Phenylalanine	45	83	88	97	86	92	43
B-Aminoisobutyric Acid	0	0	0	0	0	0	0
Homocystine	0	0	0	0	0	0	0
G-Aminobutyric Acid	0	0	0	0	0	0	0
Ethanolamine	90	108	0	65	0	0	0
Hydroxylysine	0	0	0	0	0	0	0
Ornithine	75	95	70	99	62	56	62
Lysine	153	173	155	213	135	127	138
1-Methylhistidine	7	17	6	27	21	5	5
Histidine	60	77	73	78	69	70	63
Tryptophan	36	44	50	52	47	38	25
3-Methylhistidine	5	7	5	8	6	6	5
Anserine	0	0	0	0	0	0	0
Carnosine	0	0	0	0	0	0	0
Arginine	63	49	33	55	34	32	53

Green square

Amino Acid	Baseline	Day 3	Day 5	Day 8	Day 10	Day 12	EOT
Phosphoserine	0	0	17	14	10	5	14
Taurine	36	48	44	76	48	62	38
Phosphoethanolamine	0	0	0	3	0	0	0
Aspartic Acid	0	26	37	60	23	54	10
Hydroxyproline	0	0	0	0	0	0	0
Threonine	74	68	85	244	115	147	70
Serine	88	72	80	136	89	122	72
Asparagine	34.6	0.0	0.0	0.0	0.0	0.0	26.6
Glutamic Acid	54.8	711.5	824.0	698.0	656.9	907.7	12.0
Glutamine	440.3	<12.5	<12.5	190.0	<12.5	18.5	371.9
Sarcosine	0	0	0	0	0	0	0
A-Aminiadipic Acid	0	8	0	5	0	0	6
Proline	158	97	87	311	136	253	124
Glycine	170	150	178	304	225	214	127
Alanine	347	263	298	561	328	428	216
Citrulline	24	29	24	29	24	27	12
A-Amino-n-Butyric Acid	17	17	13	22	34	26	42
Valine	263	273	281	361	284	293	327
Cystine	70	38	60	83	76	81	98
Methionine	24	15	20	48	26	31	27
Cystathionine	0	0	0	0	0	2	5
Isoleucine	64	64	64	111	67	74	86
Leucine	137	142	148	225	149	152	214
Tyrosine	94	71	69	104	70	91	87
Beta-alanine	0	0	0	0	0	0	0
Phenylalanine	72	80	90	119	122	94	132
B-Aminoisobutyric Acid	0	0	0	0	0	0	0
Homocystine	0	0	0	0	0	0	0
G-Aminobutyric Acid	0	0	0	0	0	0	0
Ethanolamine	0	64	73	71	66	80	0
Hydroxylysine	0	0	0	0	0	0	0
Ornithine	104	89	68	90	76	76	96
Lysine	167	144	160	227	127	144	156
1-Methylhistidine	0	4	19	0	9	23	4
Histidine	61	50	59	78	58	59	63
Tryptophan	40	44	46	58	40	48	25
3-Methylhistidine	0	6	7	6	6	6	5
Anserine	0	0	0	0	0	0	0
Carnosine	0	0	0	0	0	0	0
Arginine	95	43	51	81	44	60	42

Red circle

Amino Acid	Baseline	Day 3	Day 5	Day 8	Day 10	Day 12	EOT
Phosphoserine	2	0	0	0	0	2	1
Taurine	30	50	42	38	44	48	46
Phosphoethanolamine	0	0	3	0	4	4	7
Aspartic Acid	8	18	16	27	19	26	22
Hydroxyproline	0	0	0	0	0	0	0
Threonine	163	155	306	250	210	264	306
Serine	95	91	134	129	107	125	119
Asparagine	41.9	0.0	0.0	0.0	0.0	0.0	0.0
Glutamic Acid	83.1	473.8	411.5	157.6	317.4	345.1	157.9
Glutamine	583.0	195.3	266.5	406.1	254.3	227.9	443.3
Sarcosine	0	0	0	0	0	0	0
A-Aminiadipic Acid	0	0	0	0	0	0	4
Proline	177	291	296	296	304	279	157
Glycine	201	202	297	260	226	283	296
Alanine	338	470	394	358	354	322	238
Citrulline	30	25	35	23	28	24	26
A-Amino-n-Butyric Acid	20	38	27	30	27	26	39
Valine	236	249	298	268	275	226	260
Cystine	63	45	65	63	63	60	0
Methionine	18	18	23	30	22	23	17
Cystathionine	4	3	4	5	5	4	5
Isoleucine	45	68	78	77	77	70	65
Leucine	118	131	166	146	146	131	152
Tyrosine	68	71	88	97	87	76	48
Beta-alanine	0	0	0	0	0	0	0
Phenylalanine	58	112	192	110	141	167	116
B-Aminoisobutyric Acid	0	0	0	0	0	0	0
Homocystine	0	0	0	0	0	0	0
G-Aminobutyric Acid	0	0	0	0	0	0	0
Ethanolamine	0	0	0	0	0	0	0
Hydroxylysine	0	0	0	0	0	0	0
Ornithine	81	48	80	73	63	43	0
Lysine	199	142	176	208	148	145	127
1-Methylhistidine	17	20	21	38	20	19	24
Histidine	69	54	52	59	45	56	67
Tryptophan	55	57	102	93	78	61	29
3-Methylhistidine	4	5	0	0	0	0	6
Anserine	0	0	0	0	0	0	0
Carnosine	0	0	0	0	0	0	0
Arginine	61	48	73	79	54	51	37

Orange circle

Amino Acid	Baseline	Day 3	Day 5	Day 8	Day 10	Day 12	EOT
Phosphoserine	6	9	7	6	6	5	0
Taurine	37	45	28	23	28	29	52
Phosphoethanolamine	0	0	0	0	0	0	0
Aspartic Acid	6	9	20	11	18	19	15
Hydroxyproline	0	0	0	0	0	0	0
Threonine	117	109	190	268	287	331	616
Serine	88	89	119	125	135	122	236
Asparagine	41.4	0.0	0.0	0.0	0.0	0.0	0.0
Glutamic Acid	32.9	704.0	812.3	341.6	795.8	790.2	446.8
Glutamine	431.6	22.4	<12.5	278.5	<12.5	<12.5	697.7
Sarcosine	0	0	0	0	0	0	0
A-Aminiadipic Acid	0	0	0	0	6	6	0
Proline	186	189	221	187	189	183	205
Glycine	197	197	266	311	345	370	455
Alanine	293	380	531	434	280	316	360
Citrulline	23	34	32	30	32	28	35
A-Amino-n-Butyric Acid	26	38	53	48	60	38	54
Valine	216	400	389	294	299	231	332
Cystine	83	90	87	81	82	82	88
Methionine	27	28	36	29	28	24	40
Cystathionine	5	0	5	4	5	5	7
Isoleucine	63	109	100	66	80	64	93
Leucine	120	231	226	150	172	129	181
Tyrosine	86	93	118	101	102	78	68
Beta-alanine	0	0	0	0	0	0	0
Phenylalanine	82	107	124	124	132	116	85
B-Aminoisobutyric Acid	0	0	0	0	0	0	0
Homocystine	0	0	0	0	0	0	0
G-Aminobutyric Acid	0	0	0	0	0	0	0
Ethanolamine	0	0	0	0	0	0	101
Hydroxylysine	0	0	0	0	0	0	0
Ornithine	67	113	112	72	76	54	104
Lysine	227	216	273	210	169	135	216
1-Methylhistidine	0	5	3	2	2	1	1
Histidine	47	46	60	52	55	50	101
Tryptophan	65	27	28	59	36	30	27
3-Methylhistidine	0	0	0	0	8	6	7
Anserine	0	0	0	0	0	0	0
Carnosine	0	0	0	0	0	0	0
Arginine	78	60	73	58	44	37	72

	Baseline							Day 8							
**Amino Acid**	**black**	**blue**	**green**	**red**	**orange**	**Avg**	**SD**	**black**	**blue**	**green**	**red**	**orange**	**Avg**	**SD**	**P value**
Phosphoserine	0	0	0	2	6	2	2.44	5	2	14	0	6	5	5.50	0.19223
Taurine	66	52	36	30	37	44	13.18	60	68	76	38	23	53	22.10	0.47715
Phosphoethanolamine	0	0	0	0	0	0	0.00	0	0	3	0	0	1	1.18	0.34659
Aspartic Acid	6	0	0	8	6	4	3.36	16	16	60	27	11	26	19.92	**0.04114**
Hydroxyproline	0	0	0	0	0	0	0.00	0	0	0	0	0	0	0.00	--
Threonine	73	121	74	163	117	110	33.50	212	316	244	250	268	258	38.09	**0.00026**
Serine	64	75	88	95	88	82	11.19	116	136	136	129	125	128	8.30	**0.00012**
Asparagine	37.6	54.7	34.6	41.9	41.4	42	6.87	0.0	0	0.0	0.0	0.0	0	0.00	**2E-06**
Glutamic Acid	51.3	19.9	54.8	83.1	32.9	48	21.48	201.0	318	698.0	157.6	341.6	343	212.84	**0.01516**
Glutamine	487.9	670.7	440.3	583.0	431.6	523	91.48	278.5	308	190.0	406.1	278.5	292	77.51	**0.00386**
Sarcosine	0	0	0	0	0	0	0.00	0	0	0	0	0	0	0.00	--
A-Aminiadipic Acid	14	10	0	0	0	5	5.89	4	9	5	0	0	4	3.69	0.73692
Proline	151	219	158	177	186	178	23.98	204	229	311	296	187	245	55.36	**0.04037**
Glycine	157	231	170	201	197	191	25.84	211	325	304	260	311	282	46.59	**0.00596**
Alanine	267	424	347	338	293	334	53.83	263	491	561	358	434	421	115.60	0.17157
Citrulline	37	46	24	30	23	32	8.71	25	42	29	23	30	30	7.30	0.69576
A-Amino-n-Butyric Acid	19	15	17	20	26	20	3.67	36	25	22	30	48	32	10.48	**0.03285**
Valine	270	176	263	236	216	232	34.09	353	283	361	268	294	311	42.29	**0.01417**
Cystine	51	58	70	63	83	65	10.77	63	66	83	63	81	71	9.95	0.40498
Methionine	13	14	24	18	27	19	5.69	28	32	48	30	29	33	8.32	**0.01519**
Cystathionine	0	0	0	4	5	2	2.20	4	0	0	5	4	2	2.35	0.61954
Isoleucine	65	47	64	45	63	57	8.85	98	68	111	77	66	84	19.76	**0.02479**
Leucine	137	79	137	118	120	118	21.31	195	154	225	146	150	174	34.81	**0.01781**
Tyrosine	39	58	94	68	86	69	19.72	94	88	104	97	101	97	6.43	**0.02629**
Beta-alanine	0	0	0	0	0	0	0.00	0	0	0	0	0	0	0.00	--
Phenylalanine	47	45	72	58	82	61	14.30	145	97	119	110	124	119	17.73	**0.00062**
B-Aminoisobutyric Acid	13	0	0	0	0	3	5.08	0	0	0	0	0	0	0.00	0.34659
Homocystine	0	0	0	0	0	0	0.00	0	0	0	0	0	0	0.00	--
G-Aminobutyric Acid	0	0	0	0	0	0	0.00	0	0	0	0	0	0	0.00	--
Ethanolamine	50	90	0	0	0	28	36.43	0	65	71	0	0	27	37.44	0.975
Hydroxylysine	0	0	0	0	0	0	0.00	0	0	0	0	0	0	0.00	
Ornithine	96	75	104	81	67	85	13.87	95	99	90	73	72	86	12.43	0.89501
Lysine	173	153	167	199	227	184	26.43	228	213	227	208	210	217	9.65	**0.04189**
1-Methylhistidine	4	7	0	17	0	6	6.45	3	27	0	38	2	14	17.51	0.3453
Histidine	81	60	61	69	47	63	11.32	75	78	78	59	52	68	12.05	0.5547
Tryptophan	60	36	40	55	65	51	11.01	65	52	58	93	59	65	16.00	0.15945
3-Methylhistidine	6	5	0	4	0	3	2.54	7	8	6	0	0	4	3.72	0.59271
Anserine	0	0	0	0	0	0	0.00	0	0	0	0	0	0	0.00	--
Carnosine	0	0	0	0	0	0	0.00	0	0	0	0	0	0	0.00	--
Arginine	76	63	95	61	78	75	12.31	59	55	81	79	58	66	12.51	0.35188
