# Supplementary material for: Erwinia asparaginase (crisantaspase) increases plasma levels of serine and glycine
Source: Front Oncol. 2022 Dec 12;12:1035537. doi: 10.3389/fonc.2022.1035537 (PMC9790920; doi:10.3389/fonc.2022.1035537)
Supplement: Supplementary file 7 [file DataSheet_7.pdf]

Included article set

Study Number	PMID	Title	Authors	First Author	Journal/Book	Publication Year	Create Date	PMCID	NIH MS ID	DOI	Age Range (years)	Children, Adults, or both	Include participants > or =31 yo	Include participants > or = 56 yo	Malignancy	Type(s) of asparaginase studied	[Serum AA]'s measured	[Non-serum AA]'s measured
1	25348002	Pharmacokinetic and pharmacodynamic properties of calaspargase pegol Escherichia coli L-asparaginase in the treatment of patients with acute lymphoblastic leukemia: results from Children's Oncology Group Study AALL07P4	Angiolillo AL, Schore RJ, Devidas M, Borowitz MJ, Carroll AJ, Gastier-Foster JM, Heerema NA, Keilani T, Lane AR, Loh ML, Reaman GH, Adamson PC, Wood B, Wood C, Zheng HW, Raetz EA, Winick NJ, Carroll WL, Hunger SP.	Angiolillo AL	J Clin Oncol	2014	10/29/2014	PMC4239306		10.1200/JCO.2014.55.5763	1-30.99	Both	No	No	ALL	Pegylated E. coli asparaginase Calaspargase pegol	asparagine	CSF asparagine
2	18580955	Pharmacokinetic, pharmacodynamic and intracellular effects of PEG-asparaginase in newly diagnosed childhood acute lymphoblastic leukemia: results from a single agent window study	Appel IM, Kazemier KM, Boos J, Lanvers C, Huijmans J, Veerman AJ, van Wering E, den Boer ML, Pieters R.	Appel IM	Leukemia	2008	6/27/2008			10.1038/leu.2008.165	1.4-15.1	Children	No	No	ALL	Pegylated E. coli asparaginase	asparagine glutamine glutamic acid aspartic acid	All amino acids intracellularly
3	11877270	A randomized comparison of native Escherichia coli asparaginase and polyethylene glycol conjugated asparaginase for treatment of children with newly diagnosed standard-risk acute lymphoblastic leukemia: a Children's Cancer Group study	Avramis VI, Sencer S, Periclou AP, Sather H, Bostrom BC, Cohen LJ, Ettinger AG, Ettinger LJ, Franklin J, Gaynon PS, Hilden JM, Lange B, Majlessipour F, Mathew P, Needle M, Neglia J, Reaman G, Holcenberg JS, Stork L.	Avramis VI	Blood	2002	3/6/2002			10.1182/blood.v99.6.1986	1-9	Children	No	No	ALL	Pegylated E. coli asparaginase Native E. coli asparaginase	asparagine glutamine aspartic acid glutamic acid	CSF asparagine, glutamine, aspartic acid
4	8911116	Monitoring of asparaginase activity and asparagine levels in children on different asparaginase preparations	Boos J, Werber G, Ahlke E, Schulze-Westhoff P, Nowak-Gottl U, Wurthwein G, Verspohl EJ, Ritter J, Jurgens H	Boos J	Eur J Cancer	1996				10.1016/0959-8049(96)00131-1	0.1-16	Children	No	No	ALL	Native E. coli asparaginase	asparagine	

														NHL	Erwinia chrysanthemi asparaginase	aspartic acid glutamine glutamic acid	
5	7844595	Levels of L-asparagine in CSF after intramuscular administration of asparaginase from Erwinia in children with acute lymphoblastic leukemia	Dibenedetto SP, Di Cataldo A, Ragusa R, Meli C, Lo Nigro L.	Dibenedetto S	J Clin Oncol	1995	2/1/1995		10.1200/JCO.1995.13.2.339	0-14	Children	No	No	ALL	Erwinia chrysanthemi asparaginase	asparagine	CSF asparagine, glutamine, glutamate, serine, threonine, glycine, alanine, aspartate
6	21332712	l-asparaginase loaded red blood cells in refractory or relapsing acute lymphoblastic leukaemia in children and adults: results of the GRASPALL 2005-01 randomized trial	Domenech C, Thomas X, Chabaud S, Baruchel A, Gueyffier F, Mazingue F, Auvrignon A, Corm S, Dombret H, Chevallier P, Galambrun C, Huguet F, Legrand F, Mechinaud F, Vey N, Philip I, Liens D, Godfrin Y, Rigal D, Bertrand Y.	Domenech C	Br J Haematol	2011	2/22/2011		10.1111/j.1365-2141.2011.08588.x	1-55	Both	Yes	No	ALL	Native E. coli asparaginase<br>L-asparaginase encapsulated within erythrocytes (GRASPA)	asparagine	CSF asparagine
7	17132721	Pharmacodynamics and safety of intravenous pegaspargase during remission induction in adults aged 55 years or younger with newly diagnosed acute lymphoblastic leukemia	Douer D, Yampolsky H, Cohen LJ, Watkins K, Levine AM, Periclou AP, Avramis VI.	Douer D	Blood	2007	11/30/2006		10.1182/blood-2006-07-035006	17-55	Both	Yes	No	ALL	Pegylated E. coli asparaginase	asparagine	
8	29119293	Asparaginase Erwinia chrysanthemi effectively depletes plasma glutamine in adult patients with relapsed/refractory acute myeloid leukemia	Emadi A, Law JY, Strovel ET, Lapidus RG, Jeng LJB, Lee M, Blitzer MG, Carter-Cooper BA, Sewell D, Van Der Merwe I, Philip S, Imran M, Yu SL, Li H, Amrein PC, Duong VH, Sausville EA, Baer MR, Fathi AT, Singh Z, Bentzen SM.	Emadi A	Cancer Chemother Pharmacol	2018	11/10/2017		10.1007/s00280-017-3459-6	20-83	Adults	Yes	Yes	AML	Erwinia chrysanthemi asparaginase	asparagine	

																	glutamine glutamic acid	
9	8905031	L-Asparagine depletion in plasma and cerebro-spinal fluid of children with acute lymphoblastic leukemia during subsequent exposures to Erwinia L-asparaginase	Gentili D, Conter V, Rizzari C, Tschuemperlin B, Zucchetti M, Orlandoni D, D'Incalci M, Masera G.	Gentili D	Ann Oncol	1996	9/1/1996			10.1093/oxfordjournals.annonc.a010722	"Children"	Children	No	No	ALL	Erwinia chrysanthemi asparaginase	asparagine	CSF asparagine
10	15057247	Asparaginase pharmacodynamics differ by formulation among children with newly diagnosed acute lymphoblastic leukemia	Hak LJ, Relling MV, Cheng C, Pei D, Wang B, Sandlund JT, Rubnitz J, Pui CH.	Hak LJ	Leukemia	2004	4/2/2004			10.1038/sj.leu.2403351	"Children" (mean: 7.26)	Children	No	No	ALL	Native E. coli asparaginase	asparagine	CSF asparagine
																Erwinia chrysanthemi asparaginase Pegylated E. coli asparaginase		
11	26094614	A Phase 2 study of L-asparaginase encapsulated in erythrocytes in elderly patients with Philadelphia chromosome negative acute lymphoblastic leukemia: The GRASPALL/GRAALL-SA2-2008 study	Hunault-Berger M, Leguay T, Huguet F, Leprêtre S, Deconinck E, Ojeda-Uribe M, Bonmati C, Escoffre-Barbe M, Bories P, Himberlin C, Chevallier P, Rousselot P, Reman O, Boulland ML, Lissandre S, Turlure P, Bouscary D, Sanhes L, Legrand O, Lafage-Pochitaloff M, Béné MC, Liens D, Godfrin Y, Ifrah N, Dombret H; Group for Research on Adult Acute Lymphoblastic Leukemia (GRAALL).	Hunault-Berge	Am J Hematol	2015	6/23/2015			10.1002/ajh.24093	59-77	Adults	Yes	Yes	ALL	L-asparaginase encapsulated within erythrocytes (GRASPA)	asparagine	CSF asparagine
12	28244643	Plasma asparaginase activity, asparagine concentration, and toxicity after administration of Erwinia asparaginase in children and young adults with acute lymphoblastic leukemia: Phase I/II clinical trial in Japan	Ogawa C, Taguchi F, Goto H, Koh K, Tomizawa D, Ohara A, Manabe A.	Ogawa C	Pediatr Blood Cancer	2017	3/1/2017			10.1002/pbc.26475	1-25	Both	No	No	ALL	Erwinia chrysanthemi asparaginase	asparagine	
13	19741605	Comparison of native E. coli and PEG asparaginase pharmacokinetics and pharmacodynamics in pediatric acute lymphoblastic leukemia	Panetta JC, Gajjar A, Hijiya N, Hak LJ, Cheng C, Liu W, Pui CH, Relling MV.	Panetta JC	Clin Pharmacol Ther	2009	9/11/2009	PMC2831746	NIHMS175482	10.1038/clpt.2009.162	"Children"	Children	No	No	ALL	Native E. coli asparaginase	asparagine	CSF asparagine
																Erwinia chrysanthemi asparaginase Pegylated E. coli asparaginase		

14	18805963	Pharmacokinetics, pharmacodynamics, efficacy, and safety of a new recombinant asparaginase preparation in children with previously untreated acute lymphoblastic leukemia: a randomized phase 2 clinical trial	Pieters R, Appel I, Kuehnel HJ, Tetzlaff-Fohr I, Pichlmeier U, van der Vaart I, Visser E, Stigter R.	Pieters R	Blood	2008	9/23/2008		10.1182/blood-2008-04-149443	1-14	Children	No	No	ALL	Native E. coli asparaginase<br>Recombinant	asparagine<br>glutamine<br>glutamic acid	CSF asparagine
15	16434367	A pharmacological study on pegylated asparaginase used in front-line treatment of children with acute lymphoblastic leukemia	Rizzari C, Citterio M, Zucchetti M, Conter V, Chiesa R, Colombini A, Malguzzi S, Silvestri D, D'Incalci M.	Rizzari C	Haematologica	2006	1/26/2006			2-16	Children	No	No	ALL	Pegylated E. coli asparaginase	asparagine	CSF asparagine
16	10761754	L-asparagine depletion and L-asparaginase activity in children with acute lymphoblastic leukemia receiving i.m. or i.v. Erwinia C. or E. coli L-asparaginase as first exposure	Rizzari C, Zucchetti M, Conter V, Diomede L, Bruno A, Gavazzi L, Paganini M, Sparano P, Lo Nigro L, Aricò M, Milani M, D'Incalci M.	Rizzari C	Ann Oncol	2000	4/13/2000		10.1023/a:10083689168000	"Children" (median age: 4.4)	Children	No	No	ALL	Native E. coli asparaginase<br>Erwinia chrysanthemi asparaginase	asparagine	CSF asparagine
17	20545581	Silent hypersensitivity to Escherichia coli asparaginase in children with acute lymphoblastic leukemia	Strullu M, Corradini N, Audrain M, Orsonneau JL, Bouige D, Thomare P, Vermot-Desroches C, Mansuy A, Legrand A, Rozé JC, Mohty M, Méchinaud F.	Strullu M	Leuk Lymphoma	2010	6/16/2010		10.3109/10428194.2010.494316	1-17	Children	No	No	ALL	Native E. coli asparaginase	asparagine	
18	11221967	A phase I and pharmacodynamic evaluation of polyethylene glycol-conjugated L-asparaginase in patients with advanced solid tumors	Taylor CW, Dorr RT, Fanta P, Hersh EM, Salmon SE.	Taylor CW	Cancer Chemother Pharmacol	2001	2/28/2001		10.1007/s002800000207	> or = 18	Adults	Unknown	Unknown	Melanoma<br>NSCL<br>Sarcoma<br>Colon<br>Bladder<br>Cholangiocarcinoma<br>Multiple myeloma<br>Renal cell carcinoma<br>Salivary gland tumor<br>Small cell lung cancer<br>Cancer with unknown primary	Pegylated E. coli asparaginase	asparagine	

19	22961784	No evidence of increased asparagine levels in the bone marrow of patients with acute lymphoblastic leukemia during asparaginase therapy	Tong WH, Pieters R, Hop WC, Lanvers-Kaminsky C, Boos J, van der Sluis IM.	Tong WH	Pediatr Blood Cancer	2013	9/11/2012		10.1002/pbc.24292	1.8-16.4	Children	No	No	ALL	Native E. coli asparaginase	asparagine	Bone marrow asparagine, glutamine, glutamic acid, aspartic acid
																glutamine	
																glutamic acid	
																aspartic acid	
20	24449211	A prospective study on drug monitoring of PEGasparaginase and Erwinia asparaginase and asparaginase antibodies in pediatric acute lymphoblastic leukemia	Tong WH, Pieters R, Kaspers GJ, te Loo DM, Bierings MB, van den Bos C, Kollen WJ, Hop WC, Lanvers-Kaminsky C, Relling MV, Tissing WJ, van der Sluis IM.	Tong WH	Blood	2014	1/23/2014	PMC3968389	10.1182/blood-2013-10-534347	1-18	Children	No	No	ALL	Pegylated E. coli asparaginase	asparagine	
															Erwinia chrysanthemi asparaginase	glutamine	
																glutamic acid	
																aspartic acid	
21	14634792	L-Asparagine depletion levels and L-asparaginase activity in plasma of children with acute lymphoblastic leukemia under asparaginase treatment	Tsurusawa M, Chin M, Iwai A, Nomura K, Maeba H, Taga T, Higa T, Kuno T, Hori T, Muto A, Yamagata M; Japanese Children's Cancer and Leukemia Study Group.	Tsurusawa M	Cancer Chemother Pharmacol	2004	11/25/2003		10.1007/s00280-003-0734-5	"Children"	Children	No	No	ALL	Native E. coli asparaginase	asparagine	
22	23753025	Pediatric Acute Lymphoblastic Leukemia: Efficacy and safety of recombinant E. coli-asparaginase in infants (less than one year of age) with acute lymphoblastic leukemia	van der Sluis I, Möricke A, Escherich G, von Stackelberg A, Holter W, Klingebiel T, Flotho C, Legien S, Tissing W, Bierings M, Guimbal-Schmolck C, Pichlmeier U, Kühnel HJ, Pieters R.	van der Sluis I	Haematologica	2013	6/12/2013	PMC3815169	10.3324/haematol.2013.090563	<1	Children	No	No	ALL	Recombinant	asparagine	
																glutamine	
																glutamic acid	
																aspartic acid	
23	29727043	Efficacy and safety of recombinant E. coli asparaginase in children with previously untreated acute lymphoblastic leukemia: A randomized multicenter study of the Dutch Childhood Oncology Group	van der Sluis IM, de Groot-Kruseman H, Te Loo M, Tissing WJE, van den Bos C, Kaspers GJL, Bierings M, Kollen WJW, König T, Pichlmeier U, Kühnel HJ, Pieters R.	van der Sluis II	Pediatr Blood Cancer	2018	5/5/2018		10.1002/pbc.27083	1-17	Children	No	No	ALL	Native E. coli asparaginase	asparagine	CSF asparagine, glutamine, glutamic acid
															Recombinant	glutamine	
																glutamic acid	

24	7046929	Clinical evaluation of succinylated Acinetobacter glutaminase-asparaginase in adult leukemia	Warrell RP Jr, Arlin ZA, Gee TS, Chou TC, Roberts J, Young CW.	Warrell RP Jr	Cancer Treat Rep	1982	7/1/1982			"Adult"	Adults	Unknown	Unknown	ALL<br>AML<br>AMMoL<br>CML in blast phase	Succinylated Acinetobacter glutaminase-asparaginase	Glutamine<br>glutamic acid
25	19768519	The ex vivo production of ammonia predicts L-asparaginase biological activity in children with acute lymphoblastic leukemia	Watanabe S, Miyake K, Ogawa C, Matsumoto H, Yoshida K, Hirabayashi S, Hasegawa D, Inoue T, Kizu J, Machida R, Ohara A, Hosoya R, Manabe A.	Watanabe S	Int J Hematol	2009	9/22/2009		10.1007/s12185-009-0419-x	2-7	Children	No	No	ALL	Native E. coli asparaginase	asparagine

Summary of variables analyzed

Number of studies with participants in each age category

Children	17
Adults	4
Both	4
Adults≥31 yo	4
Adults≥56 yo	2

Number of studies analyzing each malignancy type

ALL	23
AML	2
AMMoL	1
CML in blast phase	1
NHL	1
Multiple myeloma	1
Melanoma	1
Salivary gland tumor	1
NSCL	1
Small cell lung	1
Cholangiocarcinoma	1
Colon	1
Renal cell carcinoma	1
Bladder	1
Sarcoma	1
Cancer with unknown primary	1

Number of studies analyzing specific asparaginases

Native E. coli asparaginase	12
Pegylated E. coli asparaginase	9
Calaspargase pegol	1
Erwinia chrysanthemi asparaginase	9
L-asparaginase encapsulated within erythrocytes (GRASPA)	2
Succinylated Acinetobacter glutaminase-asparaginase	1
Recombinant	3

Number of studies measuring specific amino acids after asparaginase therapy initiation

Asparagine	24
Glutamine	10
Glutamic acid	10
Aspartic acid	6
